# A comparison of nifedipine and tamsulosin as medical expulsive therapy for the management of lower ureteral stones without ESWL

**DOI:** 10.1038/srep05254

**Published:** 2014-06-11

**Authors:** Dehong Cao, Lu Yang, Liangren Liu, Haichao Yuan, Shenqiang Qian, Xiao Lv, Pin Han, Qiang Wei

**Affiliations:** 1 From the Department of Urology, West China Hospital, Sichuan University, Chengdu, Sichuan, China

## Abstract

Administration of nifedipine or tamsulosin has been suggested to augment stone expulsion rates. We aimed to compare the stone expulsion rates and adverse effects associated with the use of nifedipine or tamsulosin as medical expulsive therapy (MET) for the management of lower ureteral stones (LUS) without extracorporeal shock wave lithotripsy (ESWL) via a literature review and meta-analysis. Relevant randomized controlled trials (RCTs) were identified from the Medline, EMBASE, Cochrane CENTRAL, and Google Scholar databases. Finally, a total of 7 RCTs with 3897 patients were included. Our meta-analysis showed that tamsulosin could significantly increase the stone expulsion rate relative to nifedipine in patients with LUS (random-effects model; risk ratio [RR] = 0.81; 95% confidence interval [CI] = 0.75–0.88; P < 0.00001). The subgroup analysis indicated no statistically significant difference between the drugs with regard to minor or major adverse effects (fixed-effect model; RR = 1.19, 95% CI = 0.91–1.54, P = 0.20; and RR = 1.63, 95% CI = 0.22–11.82, P = 0.63, respectively). This meta-analysis demonstrated that tamsulosin was more effective than nifedipine in patients with LUS, as evidenced by the higher stone expulsion rate. Tamsulosin treatment should therefore be considered for patients with LUS.

Urolithiasis is common in the global population, affecting 1–5% of the population in Asia, 5–9% in Europe, 13% in North America, and 20% in Saudi Arabia^1^. Ureteral stones account for approximately 20% of urolithiasis cases; approximately 70% of ureteral stones are located in the lower third part of the ureter and are known as “distal ureteral stones” or “lower ureteral stones (LUS)”^2^. LUS, which are commonly encountered in urological practice, can be treated using multiple modalities^2^,^3^.

Interventional treatments comprise medical expulsive therapy (MET), extracorporeal shock wave lithotripsy (ESWL), percutaneous nephrolithotomy, ureteroscopy, laparoscopic/open stone removal, and/or a combination of these approaches. The ability of medical treatment to facilitate stone expulsion has increasingly been confirmed through research^4^,^5^,^6^,^7^. Relevant studies have greatly advanced our understanding of the role of MET in facilitating stone expulsion. Additionally, MET might also significantly reduce medical costs and prevent unnecessary surgeries and the associated risks and complications. Furthermore, patients in whom treatment failed may also choose minimally invasive treatments as auxiliary procedures. Therefore, MET for LUS has gained increasing attention in recent years.

In current practice, MET with either the nifedipine (calcium channel blocker) or the tamsulosin (alpha-receptor blocker) has been demonstrated to augment the stone passage rates of moderately sized LUS^8^. Indeed, the European Association of Urology guidelines suggest these 2 agents as reasonable treatment choices to facilitate ureteral stone expulsion^9^,^10^.

To date, no multi-national studies have compared these 2 drugs or provided overwhelming evidence regarding their benefits for LUS without ESWL. For these reasons, we conducted a systematic review and meta-analysis of the published evidence from randomized controlled trials (RCTs) to assess and compare the stone expulsion rates and adverse effects of nifedipine and tamsulosin for the management of LUS without ESWL. Seven articles were found during our search, which we have studied in an attempt to provide conclusive information in this area.

## Results

### Search results and reporting quality

**Figure 1:**
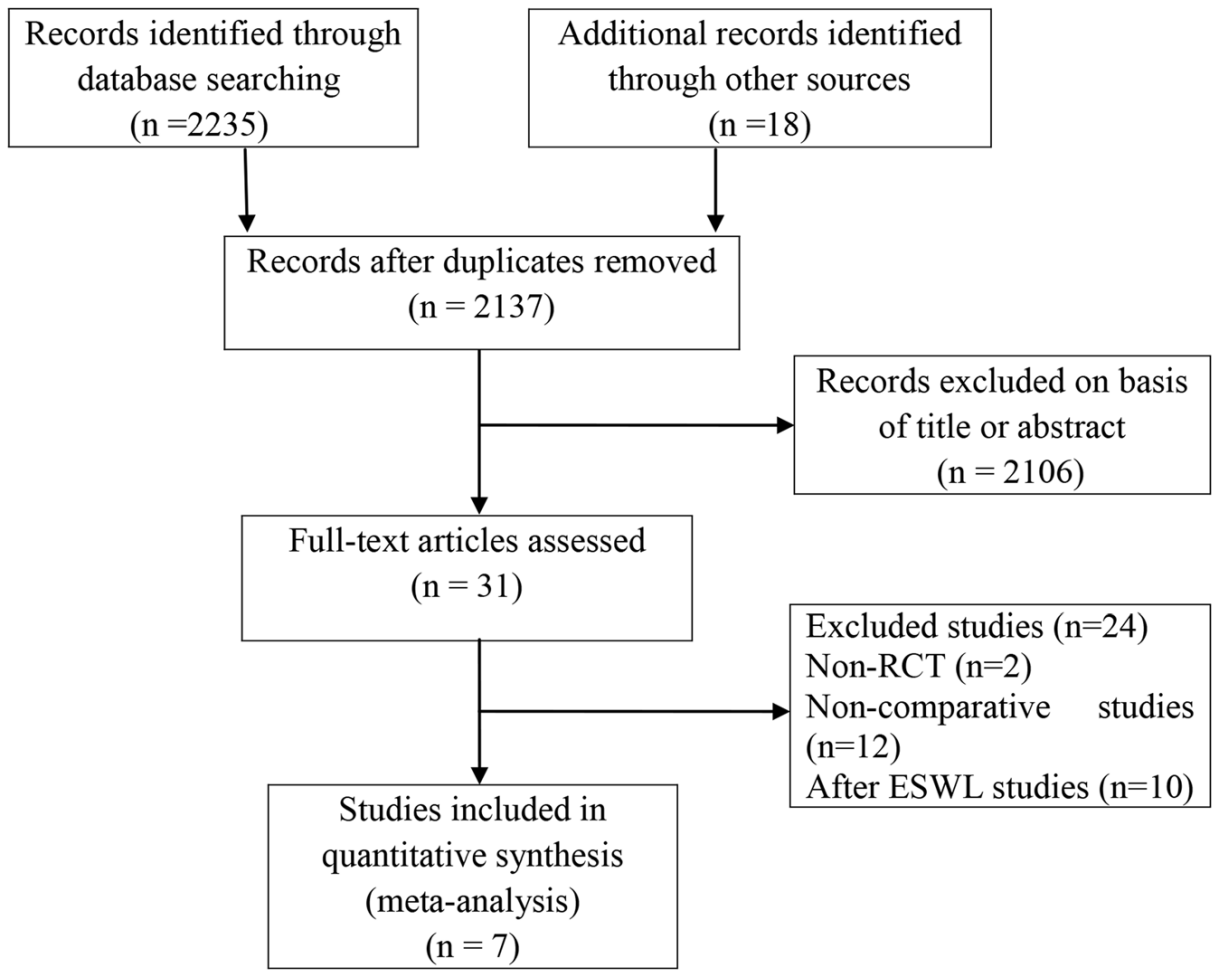
Flow diagram of evidence acquisition.

We formulated a comprehensive and exhaustive search strategy to identify all relevant studies regardless of the language or publication status. All the selected trials were RCTs; after a quality assessment, we finally included 7 RCTs identified through electronic database and manual searches^11^,^12^,^13^,^14^,^15^,^16^,^17^ in this meta-analysis. The literature screening process is summarized in Figure 1.

All eligible participants in the included trials were randomly assigned to the nifedipine or tamsulosin treatment group. A total of 3897 patients were included in the 7 study trials; of these, 1945 received nifedipine and 1952 received tamsulosin. All included studies reported the stone expulsion rates. And stone diameter greater than or equal to 3.0 mm. The included patients randomly received either 30 mg of nifedipine daily, except for the group receiving 20 mg daily in the study by Islam et al^15^, or 0.4 mg of tamsulosin daily via oral administration. The maximum treatment time for all participants was 4 weeks except for those in the trial conducted by Lü et al^13^. The basic characteristics and quality assessments of the included studies are summarized in Table 1.

### Efficacy

#### Stone expulsion rate

**Figure 2:**
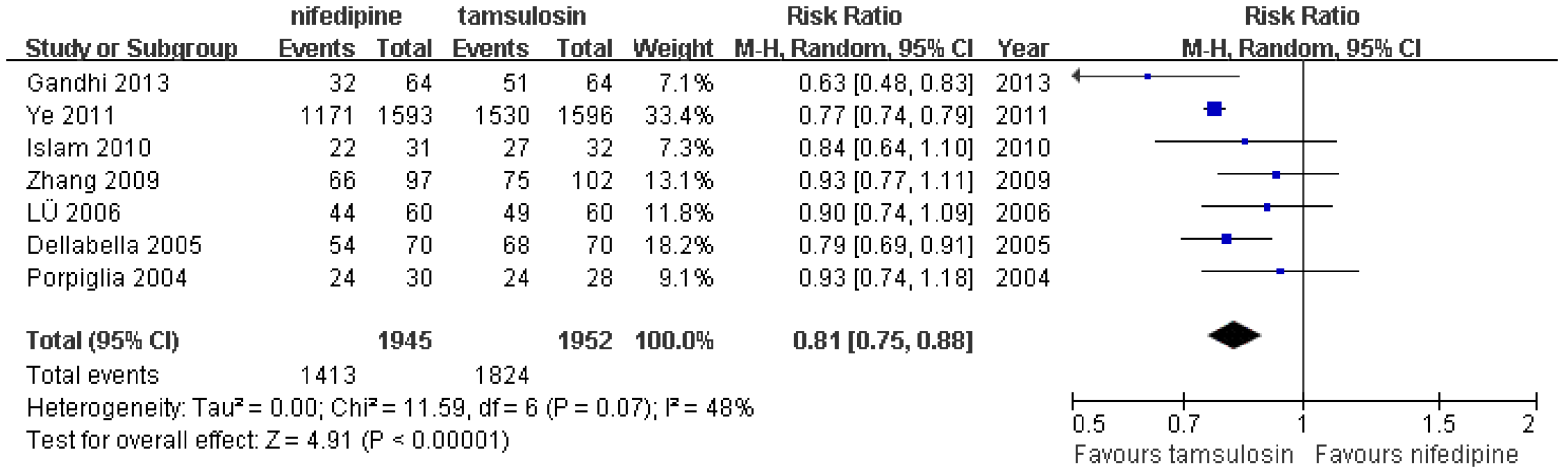
Forest plot of stone expulsion rate between nifedipine and tamsulosin group.

All 7 studies^11^,^12^,^13^,^14^,^15^,^16^,^17^ including 3,897 patients compared the stone expulsion rates between treatment groups. Moderate heterogeneity was observed in the pooled analysis (P = 0.07; *I*^*2*^ = 48%). In the random-effect model meta-analysis of the 7 studies, the pooled estimates were statistically significantly different between the 2 groups (relative risk [RR]: 0.81; 95% confidence interval [CI]: 0.75–0.88; P < 0.00001; Figure 2). This pooled analysis indicated that tamsulosin was associated with a markedly better stone expulsion rate than nifedipine. More importantly, this conclusion is stable and was not impacted by the sensitivity analysis process, which each study was sequentially excluded from the pooled analysis.

### Safety

#### Adverse effects

**Figure 3:**
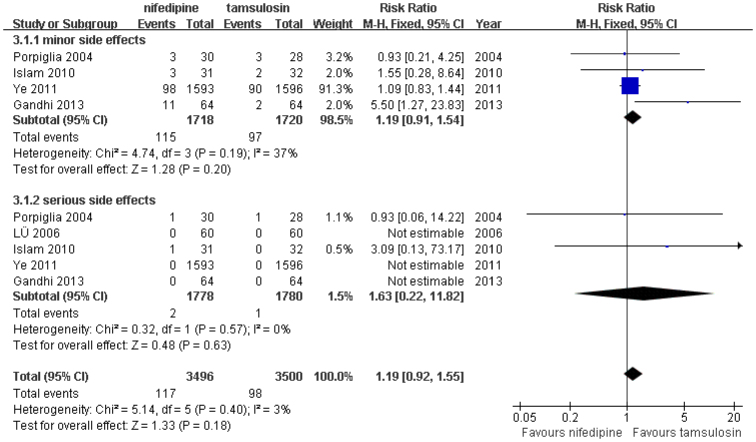
Forest plot of drug-related adverse effects between nifedipine and tamsulosin group.

Adverse effects were assessed in 5 studies^11^,^13^,^15^,^16^,^17^ that included 3,558 patients distributed between the 2 groups. A meta-analysis demonstrated no statistically significant difference between the 2 drugs in terms of the incidence of adverse effects (fixed-effects model; odds ratio [OR] = 1.19; 95% CI = 0.92–1.55; P = 0.18), and low heterogeneity was observed in the pooled analysis (P = 0.40; *I*^*2*^ = 3%). Among these 5 studies, 4^11^,^15^,^16^,^17^ that included 3,438 patients reported minor adverse effects, and no statistically significant difference was found in a subgroup analysis of the 2 groups (fixed-effects model; RR = 1.19; 95% CI = 0.91–1.54; P = 0.20). A subgroup analysis of the 5 studies that reported serious adverse effects also revealed no statistically significant difference (fixed-effects model; RR = 1.63; 95% CI = 0.22–11.82; P = 0.63; Figure 3). The subgroup analysis was stratified according to minor adverse effects and serious adverse effects. This conclusion remained stable when the subgroups were analyzed independently.

## Discussion

To our knowledge, this is an update systematic review and meta-analysis in which nifedipine and tamsulosin have been compared for the management of LUS without ESWL. Some of the previous studies and systematic reviews demonstrated that the adjuvant administration of either nifedipine or tamsulosin augmented the stone expulsion rate compared with standard therapy or a placebo in patients with upper ureteral stones treated both with and without ESWL^18^,^19^,^20^,^21^,^22^. Micali et al.^23^ reported that among patients with upper-mid ureteral stones, nifedipine was more suitable and efficacious than tamsulosin for stones after ESWL. Picozzi et al.^4^ published a systematic review and meta-analysis of MET for LUS and reported no difference between the tamsulosin and nifedipine groups with regard to the stone expulsion rate (P = 0.79). For a reliable and scientific conclusion of the comparison of nifedipine and tamsulosin for LUS without ESWL, a precise search strategy was implemented to include all comparative studies of these 2 agents. Therefore, studies of patients with ureteral stones after ESWL, non-RCTs and studies that compared either nifedipine or tamsulosin with a control were excluded from among studies of patients with LUS. Ultimately, 7 RCTs met the inclusion criteria of the present meta-analysis.

The 2 agents investigated herein have come to be more commonly used for effective management of uncomplicated LUS. These agents are thought to act by relaxing the ureteral smooth muscle through reduced intracellular calcium influx, which is modulated by the autonomic nervous system. Both nifedipine and tamsulosin appear to beneficially inhibit ureteral smooth muscle contractions that cause ureteral spasms while allowing antegrade stone propagation^24^,^25^. Although these effects are presumably dependent on the pharmacological class of these agents, only tamsulosin and nifedipine were specifically studied in the trials incorporated into this present systematic review.

The results of our meta-analysis suggested a significant improvement in the stone expulsion rate with tamsulosin relative to nifedipine for the medical management of moderately sized LUS. Although the systematic review and meta-analysis conducted by Picozzi et al.^4^ reported no difference between nifedipine and tamsulosin in facilitating LUS expulsion, our findings from the pooled analysis were consistent with those of several previous RCTs on the same subject^11^,^12^,^13^,^14^,^15^,^16^,^17^. Specifically, patients who used tamsulosin more easily passed LUS than did patients who used nifedipine.

Ureteral stone expulsion depends primarily on stone-related factors, which include size and location, as well as pathological factors, including urinary tract contraction^26^. Coll et al.^27^ revealed that ureteral stones <5 mm in size had a >75% chance of spontaneous expulsion, whereas the spontaneous expulsion rates were only 60%, 48%, and 25% for ureteral stones with sizes of 5–7 mm, 7–9 mm, and >9 mm, respectively. Similarly, stone location was also a significant factor; the spontaneous passage rates as a function of stone location were 48%, 60%, and 75% for proximal ureteral stones, mid ureteral stones, and LUS, respectively^27^. Therefore, stone size was a strong predictive factor for determining LUS expulsion, with smaller LUS having a higher chance of being expelled than larger LUS^28^. In this study, 5 RCTs included patients with stone diameters of 3.0–10.0 mm. However, Dellabella et al.^12^ included patients with stone diameters of 4.0–11.0 mm in the nifedipine group and 4.0–18.0 mm in the tamsulosin group. In addition, Gandhi et al.^17^ included patients with stone diameters of 5.0–15.0 mm for each group. Regardless, we found that tamsulosin significantly increased the stone expulsion rate relative to nifedipine, even when all literature reports were included. Therefore, in our meta-analysis, tamsulosin was associated with significantly greater stone expulsion rates than nifedipine in patients with similarly sized LUS.

Additionally, our statistical results and subgroup analysis of adverse effects demonstrated that tamsulosin was associated with slightly fewer minor and serious side effects, but this difference did not achieve statistical significance. In the present meta-analysis, the minor adverse effect incidence rates with nifedipine and tamsulosin alone were 6.7% (115/1718) and 5.6% (97/1720), respectively. Regarding reported serious adverse effects in the included studies, only 2 of 1778 patients in the nifedipine group and 1 of 1780 patients in the tamsulosin group experienced these side effects. The minor adverse effects of nifedipine or tamsulosin use, which primarily included headache, dizziness, and nausea, were tolerable and required no further medical intervention. Of the serious adverse effects, Propiglia et al.^11^ reported 1 patient with transient hypotension and palpitations in the nifedipine group and 1 patient with severe asthenia in the tamsulosin group; additionally, Islam et al.^15^ reported only 1 patient in the nifedipine group who experienced serious adverse effects associated with hypotension and palpitations. The serious adverse effects in these 3 patients disappeared following the suspension of medical therapy. The present findings agree with the results of previous clinical trials that reported similar low incidence rates of side effects with these 2 agents^11^,^12^,^13^,^14^,^15^,^16^,^17^,^22^. In other words, our meta-analysis demonstrated no statistically significant difference between these 2 drugs regarding adverse effects associated with MET for the management of LUS.

The time to stone expulsion was also reported in included studies. Picozzi et al.^4^ did not find a significant difference between the 2 different drugs with respect to the expulsion time (P = 0.17). However, many of the included studies found that tamsulosin treatment significant reduced the expulsion time relative to nifedipine. These investigators, including Dellabella et al.^12^ and Ye et al.^16^, reported median expulsion times of 5.0 and 5.7 days, respectively, with nifedipine and 3.0 and 3.3 days, respectively, with tamsulosin. Porpiglia et al.^11^, Lü et al.^13^, and Islam et al.^15^ reported average expulsion times of 9.3, 8.0, and 9.3 days, respectively, with nifedipine and 7.9, 4.0, and 7.9 days, respectively, with tamsulosin. Gandhi et al.^15^ reported median expulsion times of 23.0 days with nifedipine and 9.0 days with tamsulosin. Obviously, these studies reported shorter expulsion times with tamsulosin than with nifedipine. We thought that patients treated with tamsulosin might have a shorter expulsion time than those treated with nifedipine. Nonetheless, Large, prospective, randomized trials should be provided in the future to confirm the findings.

Our meta-analysis confirmed the positive finding that tamsulosin was superior to nifedipine with regard to stone expulsion; this finding was likely due to the higher density of alpha receptors in the lower part of the ureter^29^,^30^. Animal studies have demonstrated the effect of tamsulosin blockade on ureteral motility^31^. Based on a study by Troxel et al^25^, we also think that nifedipine blocks ureteral contraction, whereas tamsulosin significantly reduces contraction but maintains baseline activity level. Therefore, both drugs can block the disordered counteractive contractile activity associated with ureteral spasm, but the possibility that a certain degree of antegrade peristalsis might be maintained with tamsulosin could explain the slight advantage of this agent.

Our meta-analysis detected heterogeneity in the stone expulsion rate. We maintain that this heterogeneity might have resulted from differences in drug dosages and usage among the patients. Additionally, differences in the stone sizes and stone size measurement methods that included different imaging modalities could have potentially increased the degree of heterogeneity. Additional factors that have been predicted to potentially amplify heterogeneity among studies include differences in MET durations, follow-up periods, and definitions of stone expulsion success or failure.

Our systematic review and meta-analysis also has some limitations. The study quality estimation was influenced by the inadequate information provided in the publications or the methodological differences among the included studies. Some studies^12^,^13^,^14^ did not report the incidence of either minor or serious drug-related side effects. In addition, we could not obtain relevant data, which may have introduced bias. Unfortunately, the included studies also did not report the expulsion time standard deviation; hence, the statistical data could not be calculated using Review Manager (The Cochrane Collaboration, Oxford, UK).

Therefore, this systematic review and meta-analysis suggests that tamsulosin is more effective than nifedipine for the treatment of patients with LUS, as a higher rate of stone expulsion was achieved with the former in the absence of significant adverse effects. Tamsulosin treatment should therefore be considered for patients with LUS.

## Methods

### Literature search

The Medline, EMBASE, Cochrane CENTRAL, and Google Scholar databases were independently searched by 2 reviewers; this search ended in March 2014. The search used the following combined medical subject heading terms and keywords: calcium channel blockers, nifedipine; alpha antagonist, tamsulosin; medical therapy, facilitated therapy, expulsive therapy, adjunctive therapy, medical management; and LUS, lower ureteral calculi, distal ureteral stone, distal ureteral calculi. Our literature search had no language restrictions. Urology, emergency, and pharmacology-related journals were searched manually for published trials and relevant review articles.

### Selection

All relevant studies were included if they met the following criteria: (1) RCTs with all patients randomly divided into nifedipine and tamsulosin groups; (2) adult subjects with imaging diagnosis-proven unilateral, solitary LUS; (3) data for at least 1 of the pre-defined outcome measurements. Patients were excluded if they had a history of ESWL, active urinary tract infection, fever, coagulopathy, pregnancy, kidney failure, history of urinary tract surgery or endoscopic treatment, multiple stones, uncorrected distal obstruction, severe hydronephrosis, morbid obesity, or concomitant treatment with alpha adrenergic receptors blockers, calcium channel blockers, or steroids. All titles and abstracts of the included studies were independently screened by 2 authors, and full texts were reviewed when necessary. Discrepancies were resolved in consultation with Wei Q.

### Data extraction

The following information was recorded independently by 2 reviewers: the first author's name, year of publication, type of research design, intervention, total number of patients, age, and duration of follow-up. The following outcome measures were extracted from the included studies: stone expulsion rate, the main outcome defined as the complete absence of any stone based on radiologic evaluation; and adverse effects as the secondary endpoint. Minor adverse effects were defined as drug-related minor side effects such as patient-reported mild nausea, dizziness, headache, gastritis/acidity, loose stools, fatigue, flushing, palpitations, and muscle cramping. Serious adverse effects were defined as those that necessitated treatment interruption in patients who were thus unable to complete the study and included hypotension (palpitations) and severe asthenia. Discrepancies were resolved through a consensus of all authors included in this study.

### Quality assessment

The relevant data were extracted from the included studies by 2 independent reviewers, using a standardized form. The quality of the included RCTs was assessed independently by 2 reviewers according to the Jadad scale score (5 points)^32^, which evaluates studies based on randomization, blinding, and withdrawal (dropouts). A study received 1 point for each yes or 0 point for each no answer to each of those criteria. A study with a Jadad score ≥3 was considered a high-quality study^33^. Any disagreements that could not be reconciled by discussion were considered by Wei Q.

### Analysis

Review Manager (RevMan) software, version 5.1.0 was used to perform the statistical analysis. The Mantel–Haenszel chi-square test for heterogeneity and the *I*^*2*^ statistic were applied to assess heterogeneity. An *I*^*2*^ value <25% was considered to indicate low heterogeneity, a value from 25% to 50% was considered moderate heterogeneity, and a value >50%, was considered large heterogeneity. When the *I*^*2*^ value indicated significantly low heterogeneity, a fixed-effect model was applied for the meta-analysis; otherwise, a random-effect model was applied. The RR was used to assess dichotomous data. RR values with 95% CIs were calculated for the stone expulsion rates and adverse effect rates between the groups. We determined a sensitivity analysis to assess the robustness of the pooled results. Adverse effects were divided into minor side effect and serious side effect subgroups based on the degree of severity. A P-value <0.05 was considered statistically significant.

## Author Contributions

W.Q. and H.P. have contributed to the conception and design of the study; and the critical revision of the article. C.D. and Y.L. searched and selected the studies, analyzed the data, prepared figures 1–3 and drafted the article. L.L., Y.H. and Q.S. participated in the acquisition of data and statistical analysis. L.X. and C.D. participated in the interpretation of data.

## Figures and Tables

**Table 1:** Basic features and quality assessments of the included studies

Study	Year	Design	Intervention (I/C)	No. of Patients	Age (years)	Stone Size (mm)	Follow-up (weeks)	Quality levels
Porpiglia et al^11^,	2004	RCT	N 30 mg qd, max 28 days/T 0.4 mg qd max 28 days.	30/28	45.6/50.5	4.7(3.5–10.0)/5.4(3.0–10.0)	4/4	High
Dellabella et al^12^,	2005	RCT	N 30 mg qd max 28 days/T 0.4 mg qd max 28 days	70/70	41.8/43.8	6.2(4.0–11.0)/7.2(4.0–18.0)	4/4	High
Lü et al^13^,	2006	RCT	N 10 mg tid 14 days/T 0.4 mg qd max14 days.	60/60	18–53/21–55	7.0(4.0–10.0)/7.0(4.0–10.0)	2/2	High
Zhang et al^14^,	2009	RCT	N 10 mg tid/T 0.4 mg qd max 28 days	97/102	36.3/34.6	6.8(4.0–9.9)/6.9(4.0–9.9)	4/4	High
Islam et al^15^,	2010	RCT	N 20 mg qd max 28 days/T 0.4 mg qd max 28 days	31/32	47.7/46.6	6.01(3.5–10.0)/5.89(3.0–10.0)	4/4	High
Ye et al^16^,	2011	RCT	N 10 mg tid/T 0.4 mg qd max 28 days	1593/1596	22–50/18–48	5.6(4.2–6.9)/5.8(4.0–7.0)	4/4	High
Gandhi et al^17^,	2013	RCT	N 30 mg qd/T 0.4 mg qd max 28 days	64/64	18–74/18–74	8.59(5.9–15.0)/8.85(5.0–15.0)	4/4	High

RCT = randomized controlled trial; N = nifedipine slow-release oral tablets; T = tamsulosin oral treatment; I/C = intervention group (nifedipine)/control group (tamsulosin); qd = once daily; tid = thrice daily; max = maximum; vs = versus.
